# The “good enough” facilitator: elucidating the role of working alliance in the mechanism of facilitation

**DOI:** 10.1186/s43058-025-00705-0

**Published:** 2025-02-25

**Authors:** Vera Yakovchenko, Monica Merante, Matthew J. Chinman, Brittney Neely, Carolyn Lamorte, Sandra Gibson, JoAnn Kirchner, Timothy R. Morgan, Shari S. Rogal

**Affiliations:** 1 Center for Health Equity Research and Promotion, VA Pittsburgh Healthcare System, Building 30 Room 2A113, University Drive (151C), Pittsburgh, PA 15240 USA; 2 RAND Corporation, Pittsburgh, PA USA; 3 Division of Gastroenterology, Hepatology, and Nutrition, Department of Medicine, University of Pittsburgh, Pittsburgh, PA USA; 4 VA Behavioral Health Quality Enhancement Research Initiative (QUERI), Central, Arkansas Veterans Healthcare System, AR North Little Rockaq, USA; 5 Department of Psychiatry, University of Arkansas for Medical Sciences, Little Rock, AR USA; 6 VA Long Beach Healthcare System, Long Beach, CA USA; 7 Department of Medicine, University of California, Irvine, CA USA; 8 Department of Veterans Affairs, National Gastroenterology and Hepatology Program, Veterans Health Administration, Washington, DC USA; 9 Department of Surgery, University of Pittsburgh, Pittsburgh, PA USA

**Keywords:** Facilitation, Working alliance, Cirrhosis, Implementation, Veterans, Cancer surveillance, Liver, Hepatology

## Abstract

**Background:**

While facilitation is a widely used implementation strategy with proven effectiveness, the development of the facilitator-recipient relationship, i.e., working alliance, has received limited attention. However, we hypothesize that working alliance may be part of the mechanism by which facilitation activates change. This study aimed to examine the associations between working alliance, facilitation, and change in clinical care in a hybrid type 3 trial of a manualized intervention, Getting to Implementation (GTI).

**Methods:**

This concurrent triangulation mixed-methods study was conducted at 12 sites in a stepped-wedge trial. We collected surveys using the Working Alliance Inventory–Short instrument (WAI), which includes three subscales of goal alignment, task alignment, and affective bond, from three respondent types (clinical facilitator, evaluation facilitator, and site team members) after a year of intervention. Facilitation activity type and dose were tracked. Summative qualitative interviews with site champions and facilitators) elicited perceptions on working alliance, facilitation, and experiences with the intervention, and results were triangulated with statistical bivariate analyses. The associations between WAI and facilitation time, fidelity, and change in liver cancer screening rate (the primary trial outcome) were assessed.

**Results:**

Across 12 sites, facilitators and site team members completed 21 interviews and 40 WAI surveys, with site aggregate average working alliance scores of 5.9 ± 0.4 on a seven-point scale. Bond scores were highest (6.1 ± 0.5), followed by Goal (6.0 ± 0.4) and Task (5.8 ± 0.5) scores. Overall and subscale scores differed by respondent type, with site respondents consistently rating items higher than facilitators, particularly in Task items. Fidelity to the GTI process (e.g., timely completion of steps and tools) was significantly positively associated with WAI scores overall (*r* = 0.41, *p* = 0.007) and subscale scores, including Goal (*r* = 0.39, *p* = 0.011), Task (*r* = 0.42, *p* = 0.006), and Bond (*r* = 0.33, *p* = 0.039). WAI scores were not correlated with facilitation time (dose). WAI scores overall and the Bond and Goal scores were significantly positively associated with sustained improvement in cancer screening rates (*r* = 0.57, *p* = 0.015).

**Conclusions:**

In this implementation trial, working alliance between site teams and facilitators was positively associated with both fidelity and cancer screening outcomes and was notably independent of time spent providing facilitation. Findings highlight the importance of working alliance in implementation studies.

**Trial registration:**

This project was registered at ClinicalTrials.Gov (NCT04178096) on 4/29/20.

**Supplementary Information:**

The online version contains supplementary material available at 10.1186/s43058-025-00705-0.

Contributions to the literature

- This mixed-method study provides a nuanced exploration of the working alliance within facilitator-recipient relationships, a dimension underexplored in the context of facilitated interventions.
- Despite the established efficacy of facilitation, and working alliance as a potent agent of change, the intricacies of facilitator-recipient working alliance have received limited attention.
- These findings substantiate the hypothesis that a “good enough” working alliance plays a pivotal role in effecting meaningful change and advocate for heightened awareness and empirical investigation into the developmental nuances of the working alliance throughout implementation efforts.

## Background

Facilitation is a multi-faceted implementation strategy where facilitators offer problem-solving, feedback, and social and emotional support to implementation teams [1–4].While prior studies have outlined the skills required for facilitation [5], mapped facilitation mechanisms [6], including highlighting the role of external implementation support in developing team self-regulation [7], little is known about how the facilitator-recipient relationship activates change. This gap limits the optimization of facilitation as an implementation strategy.

Dyadic relationships, like those between facilitators and recipients, evolve through ongoing interaction and communication. In psychotherapy, the therapist-client relationship has long been recognized as central to therapeutic success. Early theories emphasized transference—unconscious feelings projected unto the therapist—as a mechanism of change [8]. However, contemporary work focuses on working alliance, defined by a shared mental model between individuals (typically therapist and client) on the goals and tasks of therapy, as well as the resulting bond of trust [9, 10]. High-quality working alliances enhance goal salience, surface specific tasks, foster trust, and lead to better outcomes, including emotional regulation and behavioral changes [11]. Although alliance has been applied more broadly to relationships outside psychotherapy (e.g., career counseling, education, rehabilitation, family medicine, tobacco cessation) [12], it has not been studied in implementation science.

The principles of working alliance provide a compelling parallel to implementation facilitation. Like therapists, facilitators engage in dynamic relationships with recipients to enable change. Building an effective alliance with a facilitator by fostering trust, aligning goals, and enhancing team commitment could support the adoption, implementation, and sustainment of evidence-based practices. However, empirical evidence on the role of working alliance in facilitator-recipient relationships remains limited.

This study, embedded within a larger trial of a facilitated implementation intervention for improving cirrhosis care, explores the working alliance in this new context. Liver cancer (hepatocellular carcinoma, HCC) screening rates vary significantly across care sites, ranging from 30 to 70%, despite national guidelines recommending twice annual screening [13]. Specifically, this study aimed to 1) measure and describe the working alliance between facilitators and site champions, and 2) assess the association between working alliance, facilitation, and change in cancer screening.

## Methods

### Design

This mixed methods concurrent triangulation design was applied within the context of a 12-site, stepped-wedge, hybrid type 3 study [14]. We selected this design and analytic approach to ensure quantitative and qualitative data were prioritized equally and interpreted together [15]. Quantitative data included ongoing facilitation activity tracking, post-implementation measures of working alliance, and measures of the clinical outcome (HCC screening pre and post intervention) [16]. Qualitative data included summative interviews with facilitators and site teams. The intervention ran between October 2020 and October 2022, with follow-up data collection ending in October 2023. Per regulations outlined in Veterans Health Administration (VA) Program Guide 1200.21 [17], this project was deemed a non-research operations activity. This study was registered on ClinicalTrials.gov (NCT04178096). Reporting of the implementation intervention followed Standards for Reporting Implementation Studies StaRI guidelines (see Additional file 1).

### Setting and participants

This study included 12 geographically dispersed and context diverse VA medical centers (hereafter “sites”) within the national VA healthcare system. All sites were below the national average in terms of their performance on HCC screening. Site-level approvals were obtained, and site teams were composed of a leadership-designated champion and other providers and staff across disciplines and degree/role types.

### Implementation facilitation

#### Facilitators

Each site was assigned a two-person external facilitation team: clinical expert facilitator (CF) and evaluation facilitator (EF). The CFs were three nurse practitioners, and one each physician assistant, nurse, and clinical pharmacy specialist. The two EFs were both social work trained and had undergone facilitation training from the VA Behavioral Health Quality Enhancement Research Initiative [18]. This two-facilitator approach was selected to help distribute facilitation activities and roles by specialized experience. The CFs focused on clinical/quality improvement activities, while the EFs focused on evaluation and administrative tasks. For example, EFs prepared meeting slides, updated tools, and shared materials with sites, while CFs led discussion of these materials during meetings. EFs typically fielded questions from sites and, as needed, directed sites to CFs or other resources. In assigning CFs and EFs to sites, geography and time zone was considered for familiarity with context and ease of scheduling. Both facilitators attended each predetermined biweekly meeting, with preparation preceding meetings and debriefs following meetings. In addition, all facilitators met together weekly to discuss and troubleshoot implementation issues experienced across sites. Given the novelty of this split role facilitator approach, analyses, where possible, were stratified by facilitator type.

#### Facilitation manual

The facilitation manual for this trial, “Getting to Implementation” (GTI), is a facilitator-delivered, seven-step playbook consisting of guidance and tools to help participants build teams, set goals, assess implementation barriers and facilitators, select, adapt, and plan strategies, implement while continuously evaluating and improving, and ultimately sustain a quality improvement effort [19, 20]. Each site was assigned an EF/CF pair and had hour-long, facilitated implementation meetings every other week for six months, followed by approximately monthly meetings for an additional six months.

### Data collection

#### Quantitative data

##### Facilitation tracking

Each facilitation interaction was tracked based on dose, time, mode, and primary facilitation activity [21] using time-motion tracking [22]. Facilitation activities were defined as 16 types based on Smith et al. [4]. The tracking sheet was adapted to include: 1) facilitation event categorization as outgoing or incoming to identify facilitator versus site-initiated contact, respectively; and 2) type of communication (individual or group). Simple emails (e.g., meeting invites and reminders) were assigned a standard five minutes of facilitator time, while emails with more complexity (e.g., coordinating multiple responses, content that required planning or additional work) were calculated as 15 or 30 min.

##### Fidelity tracking

We assessed three types of fidelity: facilitation, GTI process, and strategy.

##### Facilitation fidelity

The study team assigned a primary focus to each facilitation event, using one of 16 activities defined by Smith et al., and a priori coded each as pre-implementation (*n* = 8), implementation (*n* = 5), sustainment (*n* = 3) [4]. Tracking specific facilitation activities allowed for granular facilitation fidelity documentation and analysis of how facilitation was applied across sites [4].

##### GTI process fidelity

The study team assessed fidelity to the GTI process using real-time tracking of completion of pre-identified tasks and tools. Each GTI step was broken down into tasks and rated as completed, not completed, or not applicable. Step scores were summed and two cutpoints identified to create three fidelity levels (low, medium, high).

##### Strategy fidelity

For strategy fidelity, completion of a key strategy task was evaluated at pre-implementation, implementation, and sustainment phases. Each strategy was categorized as never done, doing pre-GTI, or started during GTI.

##### Working alliance

The Working Alliance Inventory-Short (WAI-S) is a validated 12-item instrument that measures agreement between two parties regarding three subscales: Goal, Task, and affective Bond [23, 24]. The WAI-S has acceptable psychometrics including on internal consistencies, inter-rater reliability, test–retest reliability, and subscale intercorrelations [12]. The Goal subscale assesses the extent to which there are collaboratively established and agreed upon goals between the dyad. The Task subscale focuses on the shared understanding of efforts and activities needed. The Bond subscale gauges confidence, appreciation, and trust towards the therapist/facilitator. Item wording was tailored for intervention relevance, and one Bond item was removed due to lack of relevance outside of the psychotherapeutic relationship. Items were scored on a seven-point Likert scale (1 = Never, 7 = Always). The WAI-S was collected from three respondent types (CF, EF, and site champion) after completion of one year of facilitated GTI.

##### Outcome

The primary outcome of reach was defined as patient-level receipt of HCC screening among Veterans with cirrhosis. This was measured using two ICD-10 diagnosis codes for cirrhosis or its complications. We determined HCC screening status using CPT codes for an ultrasound or contrasted cross-sectional imaging test within the prior six months. Veterans were included if they had cirrhosis and at least one clinical encounter in the 18 months preceding the study baseline, and assigned to the site where they had a primary care provider. If they did not have primary care assigned, they were assigned based on the site of the most clinical care. Veterans were assigned to their sites at the start of the trial and followed over time. Outcome data were collected from the VA Corporate Data Warehouse at baseline/pre-implementation, end of facilitation after 12 months, and at 24 months. These values were used to calculate site-level pre-to-post change values.

#### Qualitative interviews

We conducted semi-structured summative interviews with all 12 site teams to understand their experiences with the GTI process (e.g., “Which parts of GTI worked/didn’t work?”), strategy selection (e.g., “What made it easy/hard for you to do GTI and the strategies?”), status of barriers and facilitators (e.g., “Did the barriers/facilitators you anticipated turn out to be the right ones?”), leadership engagement and team dynamics (e.g., “Did you have capacity to do GTI and the selected strategies?”), and their relationship with the facilitators (e.g., “How has GTI team contact been?”). Facilitator interviews asked about their preparedness and experiences as a facilitator, and about impressions of the intervention (fidelity to content and process), tailoring facilitation/GTI to sites, beliefs and perceptions of the site relationship, strategy selection and tasks, and sustainment. Interviews lasted about 60 min and were recorded and transcribed verbatim.

### Analysis

First, we described facilitation in terms of dose (time spent), mode (live virtual, email, Teams instant messaging), facilitation activity type (as detailed above), and fidelity. Next, we described working alliance scores, using arithmetic means to aggregate the scores across respondents from a single site. We then used Spearman’s and Kendall’s correlation to assess bivariate associations between working alliance, facilitation time, facilitation activities, GTI fidelity, and HCC screening rates. Where possible, analysis by facilitator type (CF, EF) were conducted.

To analyze qualitative data, two coders reviewed transcripts from interviews conducted with CFs, EFs, and site champions. We developed a priori codes and used thematic analysis to analyze qualitative data and generate themes related to the WAI subscales. Finally, we came to consensus on prominent themes and triangulated data to combine, condense, and integrate quantitative and qualitative findings [25].

## Results

The mixed-methods results are structured to first present a description of participants, then the HCC screening outcome, facilitation delivered, and working alliance, along with an assessment of the associations between working alliance, facilitation, and cancer screening.

### Participant characteristics

Eight facilitators, including six CFs and two EFs, delivered GTI. Across the 12 sites, 200 individuals from sites participated in meetings or communicated (via email, telephone, or instant messaging) with GTI facilitators, of whom 53 were considered core site team members (averaging four per site, with a range of two to eight). Site participants included physicians (36%), nurses (23%), nurse practitioners (17%), clinical pharmacy specialists (9%), program support assistants (9%), and system redesign staff (2%). They represented gastroenterology (GI) (47%), internal medicine (6%), and infectious diseases (4%) departments. Participants held leadership, middle manager, and staff positions, with an average of 10 years of experience in their current job (ranging from several months to over 30 years). Interviews were conducted with 21 individuals, including 14 site champions and seven facilitators. Site champions were physicians, clinical pharmacy specialists, nurse practitioners, or nurses, primarily in GI/specialty care or internal medicine/primary care. About a quarter of site champions held leadership roles at their sites.

### Cancer screening outcome

Of the 12 sites, 11 had available HCC screening data (Site 12 was excluded due to its transition to VA’s new electronic medical record, which prevented consistent data collection and analysis). Of these 11 sites with outcome data, all had improvement in HCC screening during the implementation year (range of relative change 9.8%–59.7%), with six sites having continued improvement in the sustainment year (range of relative change −6.4%–14.3%).

### Facilitation delivery

The facilitation process (i.e., dose and mode) was defined prior to the study to include six months of twice monthly facilitation meetings, followed by six months of monthly sustainment meetings, for a total of 18–20 meetings. Facilitators delivered a total of 235 hours across 12 sites, averaging 20 ± 6 hours per site over a one-year period during 68 ± 18 facilitation events. Live virtual meetings accounted for most of these interactions (76% ± 6%, range 65%–84%), with written interactions outside of live meetings comprising 24% ± 6% (range 16%–35%). Total facilitation time was positively associated with proportion of site-initiated outreach (*r* = 0.78, *p* = 0.004).

Site teams recognized differences in the two facilitator type roles and described them in different ways. Site teams frequently praised clinical facilitators for their interpersonal qualities, connection, and genuineness, describing them as “personable,” “warm,” “patient,” “available,” “encouraging” and “easy to work with.” To establish an open, inclusive, and inviting atmosphere, facilitators incorporated tactics such as welcoming participants to calls, incorporating icebreakers, and engaging in small talk beyond the content of the project. A site participant mentioned that encouragement to have cameras on” for virtual meetings “helped every time so we had that contact, and we could really interact” (Site 10, P1). Evaluation facilitators were similarly commended for being “meticulous,” “organized and very responsive,” and “always prepared.” Their primary role involved preparing slide decks, sharing relevant materials, and addressing questions in real-time, which allowed CFs to focus on clinical facilitation.

While the GTI Playbook outlined steps, scripts were not provided to facilitators, allowing them to adapt approaches to the unique composition and needs of each team. Facilitators underscored the need to be interactive and empathize with the experiences of site teams: “We want the sites to feel like we care about what's happening on your end. It's not just about sticking to this process” (EF1). A CF explained, “We all know the right verbiage to say, but if we say it in a different format or say it in a different way…it may hit a different population more impactfully” (CF4).

### Facilitator-Site team working alliance

Across the 12 sites, 40 Working Alliance Inventory-Short (WAI) surveys were collected from facilitators (*n* = 25) and site team members (*n* = 15), resulting in a site aggregate average score of 5.9 ± 0.4 on a seven-point scale. Bond scores were highest (6.1 ± 0.5), followed by Goal (6.0 ± 0.4) and Task (5.8 ± 0.5).

Intercorrelations between WAI subscales at the individual respondent level (*n* = 40) were high and consistent with literature values: Goal x Task (0. 75 vs. 0.88 in the literature), Goal x Bond (0.79 vs. 0.84), and Task x Bond (0.73 vs. 0.79) [26]. No differences were observed in WAI or subscale scores between the three GTI rounds in aggregate or by respondent type.

### Associations between working alliance and facilitation

Working alliance scores were not significantly associated with total facilitation delivered, proportion of live facilitation, or proportion of site-initiated facilitation at the aggregate site level. However, respondent type influenced this relationship. Clinical facilitators (CFs) had significant positive associations between time spent in live meetings and the Goal (*r* = 0.66, *p* = 0.002), Task (*r* = 0.50, *p* = 0.022), and Bond (*r* = 0.48, *p* = 0.028) subscales. No similar associations were detected among evaluation facilitators (EFs) or site respondents.

The highest rated item was related to “shared goals” (6.3 ± 0.7), reflecting the centrality of goal-setting in the GTI Playbook. Sites expressed appreciation for grounding realistic goal conversations in facilitators’ prior experience and helping sites feel well equipped to meet their goals. One CF noted the importance of “setting the goal that’s attainable so that they don't feel they failed” (CF5). Part of these efforts included trying to arrange for early small wins which was perceived as an opportunity to build confidence and hope within site teams and facilitators.

The lowest rated item was in the Task subscale: “What I am doing in GTI gives me new ways of looking at cirrhosis care at my facility” (5.4 ± 1.0). Despite facilitators’ efforts to promote flexibility in solution selection, occasional challenges arose, particularly when sites exhibited “tunnel vision” (EF1). One site team member said, “It opened our mind to…different strategies that would work. At first, we were reluctant to do some strategies, but then through examination and education…we came around to certain strategies” (Site 7, P2).

Although site respondents consistently rated items higher than facilitators, only the Task subscale was significantly different between the three respondents (*p* = 0.047). For example, the item “The site believes the way we are working with their problem is correct” (CF: 5.6, EF: 5.4, Site champion: 6.1; *p* = 0.039). A key function of the facilitator was to make Tasks (i.e., improvement processes) “less overwhelming and daunting” (CF3) for sites, while internally negotiating “Should we push? Should we not push?” (CF1). Site champions echoed the value of facilitator engagement, emphasizing the importance of “talking through the process and making sure…what you're thinking and trying to implement it the right way” (Site 4, P1) to ensure successful implementation.

Fidelity to the GTI process (e.g., timely completion of steps and tools) was significantly positively associated with WAI scores overall (*r* = 0.41, *p* = 0.007) and subscale scores, including Goal (*r* = 0.39, *p* = 0.011), Task (*r* = 0.42, *p* = 0.006), and Bond (*r* = 0.33, *p* = 0.039). While sites reported a positive view of the stepwise GTI approach, both sites and facilitators recognized that sites were “itching to get going” (CF1), but this was ultimately tempered by an appreciation that “if we would have skipped any of the steps, then we could have potentially changed the outcome” (Site 2, P2). A site lead recognized the longer-term outcome and sustainability benefit of not rushing through GTI steps: “They kind of paced it for us. They wanted to make sure that we could sustain this, that we were on board with this, and that we had buy-in” (Site 2, P1).

Facilitators balanced providing guidance with encouraging autonomy, collaborating with site teams to identify, assign, and complete tasks. A CF said, “If you've done something for so long, it's hard for you to see where you're deficient, but laying out the entire process can definitely be eye opening to helping [site teams] identify those gaps and where [they] can improve*”* (CF5). A facilitator cautioned, “We want people to select their own [strategies], but I actually think we need to lead them a little bit” (CF2).

Another CF sensed occasional site frustration because of perceptions of “If you already know the answers, why are you making us go through this process?” and explained, “Because we want them to be able to see where the holes are, the gaps are, the whatever, the barriers, at their facility” (CF1). Speaking to the strength of the bond, site team members felt facilitators navigated this well, reporting that things were tailored appropriately and collaboratively: “Decisions are definitely made based on our individual facility…Because it has to be something that is doable here and realistic” (Site 1, P1). Moreover, a site lead appreciated the clinical facilitator was “energetic about the subject matter, which was helpful, and it caught on eventually” (Site 7, P1).

Facilitators engaged quiet participants and sought input beyond site champions, using silence as a facilitation tool: “that's a very hard skill to develop, talking less” (EF2). Facilitators expressed reservations about their abilities to connect with sites: “I'm trying to connect with you on a personal level and if it doesn't work, it just makes all of the interactions very challenging” (CF2). Nevertheless, an EF reflecting on the successes and challenges in the intervention, stated facilitators may be “good enough” (EF2) to address challenges and misalignments in goals, tasks, and bond to ultimately improve site screening rates. One CF reflected on the satisfaction of seeing a site achieve unexpected positive progress: “There was this ‘ah-ha’ moment, and it was so satisfying…they have things in place that they could not have dreamed for themselves, and we would not have dreamed for them before GTI” (CF2).

### Associations between working alliance and cancer screening outcome

At the site level, the Goal subscale was significantly positively associated with sustained improvement in HCC screening rates (*r* = 0.57, *p* = 0.015). Site respondents’ ratings of alliance overall (*r* = 0.50, *p* = 0.024) and Goal (*r* = 0.58, *p* = 0.008) were significantly correlated with sustained change. Site 12, which had one of the lowest alliance scores, lamented: “I'm hopeful that it's something that we can continue and build up momentum on in the future. I do think the program was very helpful to us…But I think it's going to be longer than anticipated before we see any kind of results” (Site 12, P1).

Facilitators used baseline HCC screening data to ground goals and motivate teams. Although some site members initially felt discouraged or questioned the veracity of the data, facilitators reframed these frustrations into productive dialogue: “time for venting and problem solving [may be] needed and valued” (EF1). Sites expressed a desire to “get to the work” and spend less effort on preparatory “time consuming” goal-setting activities (Site 11, P1). Despite early skepticism, goal-setting became increasingly appreciated over time: “I had a better picture once we went through the whole process” (Site 10, P1). Likewise, a CF agreed that insight about how to reach goals was only evident later: “I think sometimes landing on the ideal state is a retrospective realization” (CF2). This acknowledgment reflects the evolving nature of goal setting and the need for ongoing adaptation by sites and facilitators.

CFs’ ratings of the Bond subscale were significantly correlated with sustained changes in HCC screening rates (*r* = 0.45, *p* = 0.050). Facilitators served as a site “ally” (CF1) to empower site teams. Garnering leadership support became the most beneficial consequence of having a trusted facilitator supporting local efforts. Now, sites expressed: “It wasn't just because I wanted to. There was somebody behind me…backing pretty much everything I was doing.” (Site 3, P1). Access to local leadership was highlighted by site teams as unprecedented, which led to a feedback loop of strengthened trust, confidence, and bond with facilitators. The attention of leadership led to one site “being given the green light to do what I knew we should be doing all along” (Site 4, P1). Meanwhile, the only negative association between working alliance and the cancer screening was among EFs for the Bond subscale (*r* = −0.55, *p* = 0.026), suggesting opposing perceptions of Bond between facilitator types.

Building site team member self-efficacy was key to sustainment. While some sites expressed confidence in their ability to continue implementing independently—“[facilitators] gave us plenty of launch time” (Site 5, P1)—others remained uncertain about maintaining progress without facilitator support. Facilitators addressed these concerns by providing resources that “anticipated many of the issues that we were going to encounter” (Site 5, P1), equipping sites for long-term success.

## Discussion

Facilitation, a frequently used, evidence-based implementation strategy, is integral to implementation science. However, its underlying mechanisms remain underexplored. Our study reveals that working alliance between facilitators and site recipients may play a pivotal role in changing liver cancer screening rates, supporting the assertion that relationships are the “real agent of change” [27]. These findings reinforce the importance of future research to better understand and leverage working alliance as a foundational element in facilitation.

This study revealed that site respondents consistently rated working alliance higher than facilitators, with significant associations observed between alliance and sustained outcomes. For example, the Goal and Bond subscales correlated with sustained improvements in HCC screening, suggesting that clear, collaborative goal setting early in the implementation process paired with a meaningful development of trust fosters long-term success. These findings align with previous psychotherapy research on the prognostic value of working alliance in psychotherapy treatment outcomes [28].

Other studies have found that early alliance measures predict outcomes better than later measures [29]. Working alliance in this study was measured after implementation efforts were completed (and after most sites had obtained positive results), which could have biased the results. Site champions may have perceived a stronger alliance when their sites experienced sustained change. However, the inverse relationship between evaluation facilitators’ Bond subscale and outcomes complicates this narrative. It may be that the evaluation facilitators spent more time with or bonded more with sites that struggled more, or that different facilitator types/roles differently shaped or perceived alliance development. Future research should explore the trajectory of alliance development, including the sequence in which subscales emerge (i.e., does Bond occur earlier in implementation and precede Goal and Task?), and their relative contributions to outcomes. This could help disentangle the directionality of effect by measuring the relationship prior to seeing outcomes of the project. Moreover, measuring working alliance earlier or longitudinally could uncover patterns obscured by ceiling effects and help better understand the mechanism of these associations and how to optimize facilitation [30, 31].

Working alliance scores have practical applications in implementation. Where this evaluation of working alliance led to a perspective that facilitator’s work was “good enough” (EF2), earlier measures of working alliance scores may help adapt facilitator approaches in real-time to focus on lagging scores in Goal, Task, or Bond. Specifically, measuring alliance and reporting scores back to facilitators throughout implementation can help open dialogue for sites/recipients to raise concerns with the facilitator thereby building trust, promoting transparency, and encouraging more meaningful interactions. Future work may explore how fostering a strong working alliance early might promote engagement, receptivity, and accelerate the trajectory of improvement [29].

Facilitation is highly tailorable, and decisions on tailoring may be rooted in the perceived relationship. This parallels D.W. Winnicott’s theory of “good enough” caregiving, wherein facilitators provide substantial initial support before gradually encouraging independence [32, 33]. Teams appeared receptive to these variations in support, which may reflect a perceived attunement to their evolving needs. The fit between facilitator and recipient warrants more study, with attention to the value of and need for matching facilitator strengths with recipient needs.

Silence in therapy and facilitation both appear to contribute positively to insight and progress. In learning settings, the recommended wait times for answering a question are a minimum of three seconds, but these pauses should likely be longer in facilitation [34, 35]. Facilitators may also need ongoing training and/or clinical supervision to practice skills around discussing common challenges, generating potential solutions, and addressing silence so-as to prompt more discussion. Facilitators require a resilience to overcome the pressures of managing thoughts and feelings (personal emotional labor) while maintaining intervention integrity and guidance, accepting constructive feedback and managing multiple (often conflicting) agendas, unequal expectations, and daily responsibilities with facilitation intensity [36].

Demystifying the facilitator-recipient relationship holds practical value, especially considering the adaptability of facilitation to different contexts. Although facilitation is often described as agnostic to improvement area, facilitation may be better suited for certain settings and outcomes, and for a minimum or maximum duration.

The mode of facilitation delivery, whether virtual or in person, may influence the trajectory of relationship development, given psychotherapy tends to be more effective when delivered in person [37, 38]. While GTI was originally designed to be a rapid onsite intervention, followed by longer term virtual support, due to COVID, it was adapted to be an all-virtual format. It is unclear whether Bond would have developed differently in the context of a rapid, intense, face-to-face kickoff meeting rather than extended and ongoing facilitation interaction. Previous research on rapid improvement work in healthcare and short-term dynamic therapy has shown effectiveness, indicating that the pace of intervention is not necessarily a determinant of success [39]. Mode may also contribute to the trajectory of developing the relationship, with relationship building having more of an exponential rather than linear gain, which requires further study. We did find a strong positive association between total facilitation time and proactive outreach by sites to facilitators suggesting site comfort with facilitators encouraged more direct outreach by sites to facilitators.

GTI as a manualized approach has both benefits and drawbacks. The benefits include consistency, ease, and capacity for broader scale and spread to less expert facilitators. However, there may be some disadvantages to manualized approaches. While training therapists to apply manualized treatments leads to better technical adherence, patient outcomes have not been consistently better with such approaches and, in fact, opponents of this approach argue that manualization may inadvertently undermine positive outcomes [40]. Consistent with the psychotherapy literature, we found that sites with higher fidelity to the GTI process also scored higher on Goal and Task subscales. Teams occasionally perceived the GTI steps to be redundant, but facilitators clarified the necessity of this approach and offered flexibility in adapting meeting schedules. Occasional frustration (and recognizing frustration is the crux of change) among site teams regarding the need to complete the entire GTI process prompted deliberate effort by facilitators to help teams develop insight and uncover gaps, goals, and strategies unique to their local context.

### Strengths and limitations

This study’s novel focus on working alliance in facilitation provides a unique perspective on a widely used strategy. However, the reliance on bivariate associations and a single data collection point limits the ability to explore complex interactions or longitudinal effects. Future studies should examine larger samples, diverse settings, and the interplay between working alliance and other implementation factors.

## Conclusions

This study offers a novel examination of working alliance in the context of implementation facilitation. Working alliance may offer a mechanistic target for enabling change and thus deserves more attention. Understanding and leveraging this relationship can optimize implementation efforts, driving sustainable improvements in healthcare. Future research should prioritize refining working alliance measurement and interpretation to understand how and why facilitation implementation is (or is not) “good enough.”

## Supplementary Information

Supplementary Material 1.

## Data Availability

This quality improvement project was conducted as a non-research operations activity by the VA HIV, Hepatitis, and Related Conditions Programs (HHRC), with the stipulation that data would be presented in aggregate, given the sensitive information included in the dataset. However, interested parties can contact the Center for Health Equity Research and Promotion in the VA Pittsburgh Healthcare System (contact via Andrea.Krushinski@va.gov or shari.rogal@va.gov) for further inquiries or data requests.

## Abbreviations

CF: Clinical expert facilitator
EF: Evaluation facilitator
GTI: Getting to Implementation
HCC: Hepatocellular carcinoma
P: Participant
VA: Veterans Health Administration
WAI: Working Alliance Inventory
WAI-S: Working Alliance Inventory–Short

## References

[1] Harvey G, Kitson A. PARIHS revisited: from heuristic to integrated framework for the successful implementation of knowledge into practice. Implement Sci. 2016;11:33. 27013464 10.1186/s13012-016-0398-2 PMC4807546

[2] Ritchie MJ, Dollar KM, Miller CJ, Smith JL, Oliver KA, Kim B, et al. Using Implementation Facilitation to Improve Healthcare: Veterans Health Administration, Behavioral Health Quality Enhancement Research Initiative (QUERI). 2020. Available from: https://www.queri.research.va.gov/tools/Facilitation-Manual.pdf.

[3] Ko HC, Wang LL, Xu YT. Understanding the different types of social support offered by audience to A-list diary-like and informative bloggers. Cyberpsychol Behav Soc Netw. 2013;16(3):194–9. 23363225 10.1089/cyber.2012.0297 PMC3603495

[4] Smith JL, Ritchie MJ, Kim B, Miller CJ, Chinman MJ, Adam Kelly P, et al. Getting to Fidelity: Consensus Development Process to Identify Core Activities of Implementation Facilitation. Global Implementation Research and Applications. 2024;4(2):151–66. 10.1007/s43477-024-00119-5 PMC11100021 38765294

[5] Ritchie MJ, Parker LE, Kirchner JE. From novice to expert: a qualitative study of implementation facilitation skills. Implement Sci Commun. 2020;1:25. 32885184 10.1186/s43058-020-00006-8 PMC7427882

[6] Kilbourne AM, Geng E, Eshun-Wilson I, Sweeney S, Shelley D, Cohen DJ, et al. How does facilitation in healthcare work? Using mechanism mapping to illuminate the black box of a meta-implementation strategy. Implement Sci Commun. 2023;4(1):53. 37194084 10.1186/s43058-023-00435-1 PMC10190070

[7] Roppolo RH, McWilliam J, Aldridge WA 2nd, Jenkins RH, Boothroyd RI, Moore LR. Is the Concept of Self-Regulation Useful for Supporting Effective Implementation in Community Settings? Clin Child Fam Psychol Rev. 2019;22(1):118–28. 30761434 10.1007/s10567-019-00286-0

[8] Freud S, Breuer J. Studies in hysteria. Luckhurst N, Trans. New York: Penguin Press; 2004.

[9] Ardito RB, Rabellino D. Therapeutic alliance and outcome of psychotherapy: historical excursus, measurements, and prospects for research. Front Psychol. 2011;2:270. 22028698 10.3389/fpsyg.2011.00270 PMC3198542

[10] Horvath AO, Del Re A, Flückiger C, Symonds D. Alliance in individual psychotherapy. Psychotherapy. 2011;48(1):9. 21401269 10.1037/a0022186

[11] Baier AL, Kline AC, Feeny NC. Therapeutic alliance as a mediator of change: A systematic review and evaluation of research. Clin Psychol Rev. 2020;82: 101921. 33069096 10.1016/j.cpr.2020.101921

[12] Paap D, Karel Y, Verhagen AP, Dijkstra PU, Geertzen JHB, Pool G. The Working Alliance Inventory’s Measurement Properties: A Systematic Review. Front Psychol. 2022;13: 945294. 35910993 10.3389/fpsyg.2022.945294 PMC9337219

[13] Heimbach JK, Kulik LM, Finn RS, Sirlin CB, Abecassis MM, Roberts LR, et al. AASLD guidelines for the treatment of hepatocellular carcinoma. Hepatology. 2018;67(1):358–80. 28130846 10.1002/hep.29086

[14] Rogal SS, Yakovchenko V, Morgan T, Bajaj JS, Gonzalez R, Park A, et al. Getting to implementation: a protocol for a Hybrid III stepped wedge cluster randomized evaluation of using data-driven implementation strategies to improve cirrhosis care for Veterans. Implement Sci. 2020;15(1):92. 33087156 10.1186/s13012-020-01050-7 PMC7579930

[15] Hanson WE, Creswell JW, Clark VLP, Petska KS, Creswell JD. Mixed methods research designs in counseling psychology. J Couns Psychol. 2005;52(2):224–35.

[16] Bidassie B, Williams LS, Woodward-Hagg H, Matthias MS, Damush TM. Key components of external facilitation in an acute stroke quality improvement collaborative in the Veterans Health Administration. Implement Sci. 2015;10:69. 25971405 10.1186/s13012-015-0252-y PMC4437451

[17] Department of Veterans Affairs Office of Research & Development. Program Guide 1200.21: VHA Operations Activities That May Constitute Research. 2019. https://www.research.va.gov/resources/policies/ProgramGuide-1200-21-VHA-Operations-Activities.pdf. Accessed June 21, 2024.

[18] Kirchner JE, Dollar KM, Smith JL, Pitcock JA, Curtis ND, Morris KK, et al. Development and preliminary evaluation of an implementation facilitation training program. Implementation Research and Practice. 2022;3:26334895221087476. 37091085 10.1177/26334895221087475 PMC9924286

[19] Rogal SS, Jonassaint C, Ashcraft L, Freburger J, Yakovchenko V, Kislovskiy Y, et al. Getting To Implementation (GTI)-Teach: A seven-step approach for teaching the fundamentals of implementation science. J Clin Transl Sci. 2022;6(1): e100. 36106128 10.1017/cts.2022.420 PMC9428668

[20] Yakovchenko V, Rogal SS, Goodrich DE, Lamorte C, Neely B, Merante M, et al. Getting to implementation: Adaptation of an implementation playbook. Front Public Health. 2022;10: 980958. 36684876 10.3389/fpubh.2022.980958 PMC9853037

[21] Ritchie MJ, Kirchner JE, Townsend JC, Pitcock JA, Dollar KM, Liu CF. Time and Organizational Cost for Facilitating Implementation of Primary Care Mental Health Integration. J Gen Intern Med. 2020;35(4):1001–10. 31792866 10.1007/s11606-019-05537-y PMC7174254

[22] Kim B, Miller CJ, Ritchie MJ, Smith JL, Kirchner JE, Stolzmann K, et al. Time-motion analysis of external facilitation for implementing the Collaborative Chronic Care Model in general mental health clinics: Use of an interval-based data collection approach. Implement Res Pract. 2022;3:26334895221086276. 37091094 10.1177/26334895221086275 PMC9924237

[23] Hatcher RL, Gillaspy JA. Development and validation of a revised short version of the working alliance inventory. Psychother Res. 2006;16(1):12–25.

[24] Busseri MA, Tyler JD. Interchangeability of the Working Alliance Inventory and Working Alliance Inventory. Short Form Psychol Assess. 2003;15(2):193–7. 12847779 10.1037/1040-3590.15.2.193

[25] Braun V, Clarke V. Using thematic analysis in psychology. Qual Res Psychol. 2006;3(2):77–101.

[26] Horvath AO. An exploratory study of the working alliance: Its measurement and relationship to therapy outcome: University of British Columbia; 1981.

[27] Yalom ID. The gift of therapy: An open letter to a new generation of therapists and their patients. New York: Harper Collins; 2002.

[28] Murphy R, Hutton P. Practitioner Review: Therapist variability, patient-reported therapeutic alliance, and clinical outcomes in adolescents undergoing mental health treatment - a systematic review and meta-analysis. J Child Psychol Psychiatry. 2018;59(1):5–19. 28681928 10.1111/jcpp.12767

[29] Zilcha-Mano S, Errazuriz P. Early development of mechanisms of change as a predictor of subsequent change and treatment outcome: The case of working alliance. J Consult Clin Psychol. 2017;85(5):508–20. 28345940 10.1037/ccp0000192

[30] Meier ST, Feeley TH. Ceiling effects indicate a possible threshold structure for working alliance. J Couns Psychol. 2022;69(2):235. 34292029 10.1037/cou0000564

[31] Horvath AO, Luborsky L. The role of the therapeutic alliance in psychotherapy. J Consult Clin Psychol. 1993;61(4):561. 8370852 10.1037//0022-006x.61.4.561

[32] Winnicott DW. Transitional Objects and Transitional Phenomena: A Study of the First Not-Me Possession. Int J Psychoanal. 1953;34:89–97. 13061115

[33] Winnicott DW. Primary maternal preoccupation. In: Mariotti P, editor. The maternal lineage: Identification, desire, and transgenerational issues. 1st ed. London: Routledge; 2012. p. 59–66.

[34] Knol ASL, Koole T, Desmet M, Vanheule S, Huiskes M. How speakers orient to the notable absence of talk: a conversation analytic perspective on silence in psychodynamic therapy. Front Psychol. 2020;11: 584927. 33364999 10.3389/fpsyg.2020.584927 PMC7750524

[35] Pfeifer E, Wittmann M. Waiting, thinking, and feeling: variations in the perception of time during silence. Front Psychol. 2020;11:602. 32300325 10.3389/fpsyg.2020.00602 PMC7142212

[36] Olmos-Ochoa TT, Ganz DA, Barnard JM, Penney L, Finley EP, Hamilton AB, et al. Sustaining implementation facilitation: a model for facilitator resilience. Implement Sci Commun. 2021;2(1):65. 34154670 10.1186/s43058-021-00171-4 PMC8218441

[37] Alldredge CT, Burlingame GM, Yang C, Rosendahl J. Alliance in group therapy: A meta-analysis. Group Dyn Theory Res Pract. 2021;25(1):13.

[38] Fluckiger C, Del Re AC, Wampold BE, Horvath AO. The alliance in adult psychotherapy: A meta-analytic synthesis. Psychotherapy (Chic). 2018;55(4):316–40. 29792475 10.1037/pst0000172

[39] Anderson EM, Lambert MJ. Short-term dynamically oriented psychotherapy: A review and meta-analysis. Clin Psychol Rev. 1995;15(6):503–14.

[40] Addis ME, Cardemil EV, Duncan BL, Miller SD. Does Manualization Improve Therapy Outcomes? In: Norcross JC, Beutler LE, Levant RF, editors. Evidence-based practices in mental health: Debate and dialogue on the fundamental questions. Washington, DC: American Psychological Association; 2006. p. 131–60.

